# Preventing lung pathology and mortality in rabbit *Staphylococcus aureus* pneumonia models with cytotoxin-neutralizing monoclonal IgGs penetrating the epithelial lining fluid

**DOI:** 10.1038/s41598-019-41826-6

**Published:** 2019-03-29

**Authors:** Lukas Stulik, Harald Rouha, Delphine Labrousse, Zehra Claire Visram, Adriana Badarau, Barbara Maierhofer, Karin Groß, Susanne Weber, Miroslava Dominis Kramarić, Ines Glojnarić, Gábor Nagy, Delphine Croisier, Eszter Nagy

**Affiliations:** 1 Arsanis Biosciences, Vienna, Austria; 2 Vivexia, Dijon, France; 3 Fidelta Ltd, Zagreb, Croatia

## Abstract

*Staphylococcus aureus* pneumonia is associated with high mortality irrespective of antibiotic susceptibility. Both MRSA and MSSA strains produce powerful cytotoxins: alpha-hemolysin(Hla) and up to five leukocidins – LukSF-PV, HlgAB, HlgCB, LukED and LukGH (LukAB) – to evade host innate defense mechanisms. Neutralizing cytotoxins has been shown to provide survival benefit in rabbit *S. aureus* pneumonia models. We studied the mechanisms of protection of ASN100, a combination of two human monoclonal antibodies (mAbs), ASN-1 and ASN-2, that together neutralize Hla and the five leukocidins, in rabbit MRSA and MSSA pneumonia models. Upon prophylactic passive immunization, ASN100 displayed dose-dependent increase in survival and was fully protective against all *S. aureus* strains tested at 5 or 20 mg/kg doses. Macroscopic and microscopic lung pathology, edema rate, and bacterial burden were evaluated 12 hours post infection and reduced by ASN100. Pharmacokinetic analysis of ASN100 in bronchoalveolar-lavage fluid from uninfected animals detected efficient penetration to lung epithelial lining fluid reaching peak levels between 24 and 48 hours post dosing that were comparable to the mAb concentration measured in serum. These data confirm that the ASN100 mAbs neutralize the powerful cytotoxins of *S. aureus* in the lung and prevent damage to the mucosal barrier and innate immune cells.

## Introduction

*Staphylococcus aureus* is one of the most common pathogens causing nosocomial infections, including hospital- and health-care associated pneumonia (HAP or HCAP), and ventilator-associated pneumonia (VAP)^1,2^. *S. aureus* also causes community-acquired pneumonia (CAP) and its most severe form, necrotizing pneumonia, which is characterized by a sudden onset, rapid worsening of symptoms, leukopenia, airway hemorrhages, severe respiratory failure, and high mortality^3^. Necrotizing pneumonia is frequently associated with community-acquired methicillin-resistant *S. aureus* (CA-MRSA) strains expressing the Panton-Valentine Leukocidin (PVL or LukSF-PV), a bi-component pore-forming cytotoxin^4^. The role of LukSF-PV in necrotizing pneumonia has been controversial due to conflicting results from different disease models^5^. Mutant *S. aureus* strains lacking *lukSF-PV* did not show an altered virulence phenotype in rodents or non-human primates^6–10^, while greatly reduced virulence was observed in rabbit pneumonia models^11^. This had been enigmatic until it was revealed that phagocytes from mice and cynomolgus monkeys are resistant to LukSF-PV, while human and rabbit phagocytes are highly sensitive to this leukocidin^12–14^.

The *lukSF-PV* gene is present in only approximately 5 to 10% of *S. aureus* isolates, therefore the majority of *S. aureus* pneumonia cases (that are non-necrotizing type) are caused by *lukSF-PV* negative strains^15,16^. In addition to LukSF-PV, four other bi-component leukocidins – two gamma-hemolysins (HlgAB and HlgCB), LukED, and LukGH (also known as LukAB) – are produced by *S. aureus*. All of them are able to lyse human phagocytes^17,18^. *lukED* is present in approximately 60% of *S. aureus* strains, while *hlgACB* and *lukGH* are part of the core genome^17,18^. Alpha-hemolysin (Hla), the best characterized cytotoxin of *S. aureus*, is an important virulence factor encoded in the core genome that has been shown to have a major contribution to pneumonia pathogenesis in all animal models tested^6,19–22^. Hla forms a very similar pore structure as the bi-component leukocidins (comprised of polymers of S- and F-components), although assembled from one type of component^18,23^. It disrupts the physical barriers in the lung by lysing epithelial and endothelial cells and therefore promotes bacterial invasion from the mucosal surface to the lung tissue and the blood stream^23^. Hla is lytic to rabbit but not to human red blood cells, explained by the lack of ADAM-10 expression on the latter^24^.

We previously showed that passive immunization with a monoclonal antibody (mAb), ASN-1,which neutralizes Hla, LukSF-PV, HlgAB, HlgCB, and LukED, fully prevented mortality in a *lukSF-PV* positive USA300 CA-MRSA pneumonia model in rabbits, while neutralizing only Hla resulted in a significantly lower effect (25% survival)^19^. Similar data were reported by others with another Hla-specific mAb (33% survival) in the same rabbit model^20^. Importantly, Hla-specific mAbs are fully protective in murine or ferret models of *S. aureus* pneumonia^19,20^. These data suggest that, unlike in rodents, bi-component leukocidins are important in pneumonia pathogenesis in rabbits. This is explained by the insensitivity of rodent phagocytic cells not only to LukSF-PV, but also to the other leukocidins^14,19,23^. Based on this species specificity of the leukocidins, their role in pneumonia pathogenesis cannot be studied in mice, rats, or ferrets. In this respect, rabbits represent the most suitable model organism, as similar to humans, their phagocytes can be lysed by all five leukocidins that human *S. aureus* isolates may express^11,14,19,25^.

Due to the high mortality in *S. aureus* pneumonia - irrespective of whether it is caused by MSSA or MRSA - alternative or adjunct therapeutic modalities are needed. One possible approach is the silencing of major virulence factors, such as Hla and the bi-component leukocidins, using human mAbs. The aim of passive immunization is to support host innate defense mechanisms by preserving mucosal integrity and viability of phagocytic cells, both of which are cornerstones in fending off *S. aureus*. The challenge for such an approach is the efficient neutralization of all these cytotoxins with a limited number of mAbs. We have developed two fully human mAbs (ASN-1 and ASN-2) that together neutralize Hla and the five bi-component cytotoxins HlgAB, HlgCB, LukSF-PV, LukED and LukGH, thereby achieving this goal^26–28^. The combination of the two mAbs, ASN100, was tested in a Phase 2 clinical trial (NCT02940626; data are currently being analyzed) enrolling mechanically ventilated patients who are heavily colonized by *S. aureus* in their lower airways, with the aim to prevent progression to pneumonia.

In this study, we evaluated the prophylactic efficacy of ASN100 in lethal rabbit pneumonia models using different *S. aureus* strains representing both MRSA and MSSA, with and without LukSF-PV expression. We also characterized the effects of different protective doses of ASN100 on tissue integrity, bacterial burden, and white blood cell counts in the lung early during infection. Quantification of the ASN100 mAbs in the rabbit airway epithelial lining fluid (ELF) was performed to study their pharmacokinetics and penetration into the lung tissue, as such data is scarce, and little is known about IgG1 levels in pulmonary mucosal fluids.

## Results

### ASN100 improves survival in rabbit MRSA and MSSA pneumonia models

We established lethal pneumonia models in rabbits by intra-tracheal instillation of four different challenge strains representing USA300 CA-MRSA, USA100 HA-MRSA, and two MSSA strains with different sequence (ST) and *spa*-types: ST72-t148 and ST152-t3621. The USA300 CA-MRSA and the ST152 MSSA strains carried *lukSF-PV*, and all four strains were tested positive for *hla*, *hlgACB*, *lukED* and *lukGH* by PCR (data not shown). Immunoblot analysis of culture supernatants of the four types of *S. aureus* strains grown in three different growth media confirmed expression of Hla by all strains, and LukSF-PV by the two *lukSF-PV* positive strains. For the other leukocidins, we observed different expression levels, for example the USA100 HA-MRSA strain produced overall the lowest levels of leukocidins and Hla, while the ST72 MSSA produced the highest levels of LukD (Fig. S1).

In pilot experiments, rabbits were infected with different bacterial inoculum sizes; the minimal lethal inoculum was between approximately 1.5 to 9 × 10^9^ CFU/rabbit, resulting in 100% lethality within 24 hours post challenge (data not shown).

**Figure 1:**
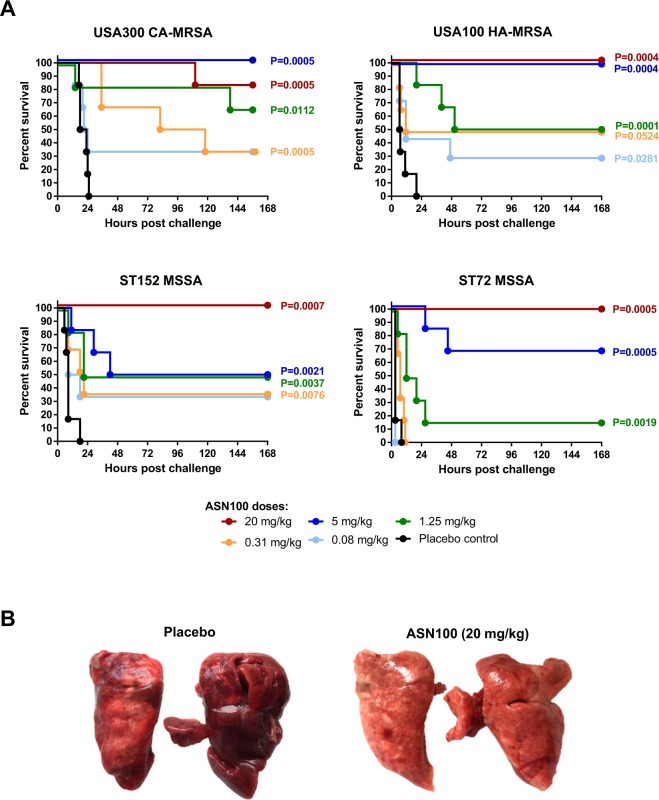
Dose-dependent protection elicited by ASN100 in rabbit models of lethal pneumonia. (**A**) Rabbits were intravenously immunized with ASN100 (0.08 mg/kg to 20 mg/kg) or placebo (ASN100 formulation buffers) 24 hours before intra-tracheal challenge with *S. aureus* strains indicated. Survival was monitored for 168 hours post-challenge. Results of two independent experiments are shown (each with 3 rabbits/group, total n = 6). Kaplan-Meier plots show survival of animals including statistical analysis (log-rank, Mantel-Cox) indicated as P-values relative to the placebo control group. (**B**) Representative pictures of lungs from infected rabbits challenged with the USA300 CA-MRSA (LAC) strain that received ASN100 (20 mg/kg) or placebo 24 hours before. Pictures taken at time of death (18 hours; placebo) or at the end of the study (7 days; ASN100).

To assess the *in vivo* efficacy of ASN100 in preventing pneumonia-associated death, rabbits were passively immunized with single intravenous doses of an equimolar mixture of ASN-1 and ASN-2 (in the range of 0.08 to 20 mg/kg total mAb), 24 hours prior to challenge with lethal inocula of the different *S. aureus* strains. We found that the highest ASN100 dose tested (20 mg/kg) prevented death in all four models (except one animal that succumbed more than 96 hours after challenge, outside the acute phase of infection in the USA300 CA-MRSA model (Fig. 1A). While the 5 mg/kg dose was still fully protective against the two MRSA strains, the survival rates in the two MSSA models decreased to 50 or 67%. Lower doses elicited variable, but still significant protection in the different models. Subsequently, mAb doses between 5 and 20 mg/kg were also tested with the ST72 MSSA strain and the minimal fully protective dose was found to be 10 mg/kg (data not shown).

Macroscopic examination of the lungs revealed severe necrosis and hemorrhage in control animals that was prevented or greatly reduced by ASN100 (example shown in Fig. 1B).

### Cytotoxin neutralization inhibits *S. aureus* pathogenesis at early stage of infection

The rapid course of these models, with animals succumbing within 24 hours after *S. aureus* challenge, is suggestive for a severe pathology developing early during infection. To gain insight into these early events and to investigate the effect of cytotoxin neutralization, we sacrificed rabbits challenged with a USA300 CA MRSA strain 12 hours after infection when all animals, including controls, were alive. The control group was compared to animals treated with 1.25, 5, or 20 mg/kg of ASN100, 24 hours before induction of lethal pneumonia.

**Figure 2:**
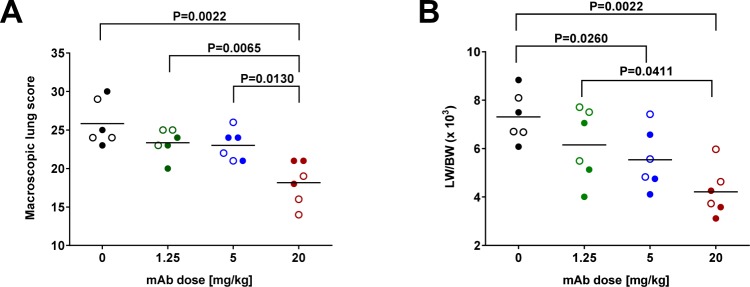
Prophylaxis by ASN100 improves the macroscopic lung pathology in necrotizing pneumonia. 24 hours prior to lethal intra-tracheal challenge with the *S. aureus* LAC strain rabbits intravenously immunized with ASN100 at indicated doses or placebo. Macroscopic lung tissue scores (**A**) and lung edema rates (LW/BW × 10^3^) (**B**) of rabbits treated with ASN100 (1.25, 5, or 20 mg/kg) or placebo, evaluated at 12 hours post challenge. Individual animals are represented by open and closed symbols for the two independent studies and lines indicate the group mean values. P-values were calculated based on group-comparison by the Mann-Whitney test and are shown for all statistically different comparisons.

The cumulative pulmonary macroscopic scores of individual lobes demonstrated an ASN100 dose-dependent reduction in lung pathology (Fig. 2A). The highest dose was associated with significantly lower disease scores, while at lower doses partial effects were seen, even with the 5 mg/kg dose that was fully protective in the survival readout. Similarly, a dose-dependent improvement of lung edema rates was observed, most prominently with the highest ASN100 dose (Fig. 2B).

**Figure 3:**
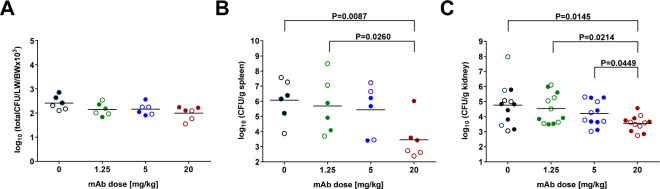
Effect of ASN100 on bacterial lung-burden and systemic dissemination. 24 hours prior to lethal challenge with the *S. aureus* LAC strain, rabbits were intravenously immunized with ASN100 (1.25, 5, or 20 mg/kg) or placebo. Bacterial organ loads were determined from lungs (**A**), spleens (**B**), left and right kidneys (**C**), by plating of homogenized organ samples harvested at 12 hours post challenge. Individual animals are represented by open and closed symbols for the two independent studies (each with 3 rabbits/group) and lines indicate the group mean values. P-values were calculated based on group-comparison by the Mann-Whitney test and are shown for all statistically different comparisons.

Importantly, significantly lower bacterial burden was detected in the lung, spleen, and kidneys of animals treated with the highest dose of ASN100, while with the two lower mAb doses, a tendency for reduction was seen (Fig. 3).

**Figure 4:**
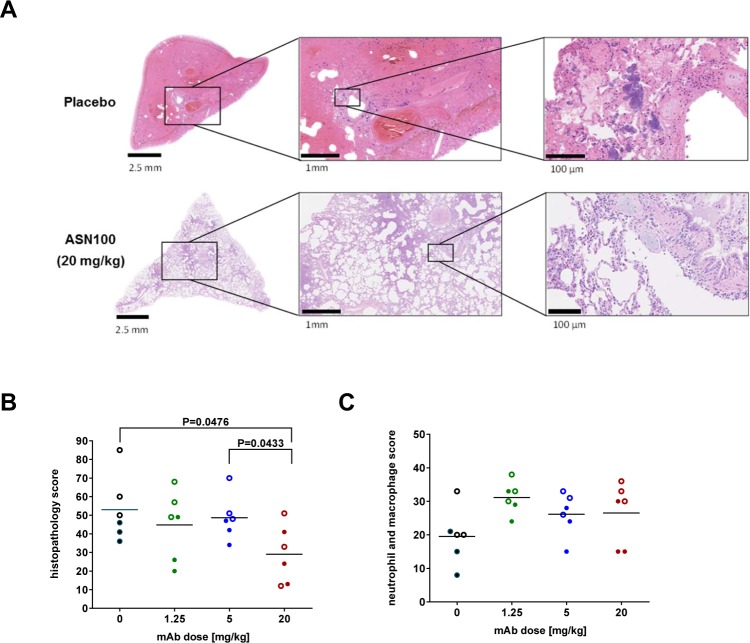
ASN100 improves the histological lung pathology and immune-cell counts in necrotizing pneumonia. 24 hours prior to lethal intra-tracheal challenge with the *S. aureus* LAC strain, rabbits were intravenously immunized with ASN100 (1.25, 5, or 20 mg/kg) or placebo. Animals were sacrificed 12 hours post challenge and sections of each pulmonary lobe were stained by H&E. (**A**) Representative sections of the central pulmonary lobes (with respective magnifications of areas of interest) of rabbits receiving either placebo or ASN100 (20 mg/kg). Cumulative sum of the lung tissue damage (**B**) as well as neutrophil and macrophage counts (**C**), based on semi-quantitative histopathological qualifiers which was scored for each pulmonary lobe. Individual animals are represented by open and closed symbols for two independent studies and lines indicate the group mean values. P-values were calculated based on group-comparison by the Mann-Whitney test and are shown for all statistically different comparisons.

Microscopic analysis of hematoxylin-eosin stained lung tissues revealed severe pathology in all pulmonary lobes of control animals. The main alteration was multifocal coalescing lesions centered on alveolar spaces that were extended and filled with proteinaceous fluid (edema), fibrin exudation forming proteinaceous casts and hyaline membranes, and hemorrhage. The lung parenchyma, mainly the alveolar septa, showed diffuse moderate congestion of arterioles and capillaries. Pneumocytes were often lost. Epithelial cells of the bronchi and bronchioles were degenerated, desquamated, and replaced by fibrin and suppuration (examples are shown in Fig. 4A). We found that the highest dose of ASN100 significantly improved lung tissue integrity. This was also supported by the lower semi-quantitative disease score that was again significantly improved with the highest ASN100 dose, but not with the lower doses (Fig. 4B). There was a tendency for increased numbers of neutrophils and macrophages in the lungs of ASN100 treated animals (at any dose), determined in stained tissues (Fig. 4C).

Spleens showed minimal to mild activation of the red and white pulp (data not shown) with the lowest activation score observed in the 20 and 5 mg/kg ASN100 groups (score of 0.7 in both groups) in comparison to the control group (score of 1.3). No significant pathology was observed in the kidneys at this early time point post-infection.

### Kinetics of monoclonal antibody penetration into the lung

As limited information is available for the penetration of human IgG1 mAbs into the lung in general, and in rabbits in particular, we determined the concentration of ASN-1 and ASN-2 in bronchoalveolar lavage fluid (BALF) at different time points after intravenous administration of 40 mg/kg ASN100 and compared to those measured in the serum. ASN-1 and ASN-2 mAb levels were determined by ELISA using anti-idiotype antibodies recognizing the complementarity-determining regions (CDRs), therefore detecting “free” mAbs not engaged in antigen binding. In parallel, anti-human and anti-rabbit IgG reagents were also used in ELISA to measure total human and rabbit IgG levels. Urea concentrations of BALF and serum samples were determined to normalize for the dilution of BALF samples and to calculate the real IgG concentrations in the epithelial lining fluid (ELF) of the single matched time point samples^29–31^.

**Figure 5:**
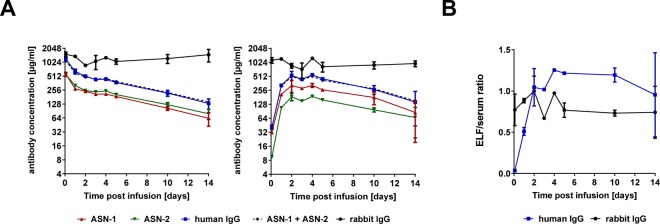
Pharmacokinetics of ASN-1 and ASN-2 in serum and ELF of uninfected rabbits. Concentrations of ASN-1 and ASN-2, as well as total human IgG and total rabbit IgG were determined in serum and ELF of uninfected rabbits immunized with 40 mg/kg of ASN100. (**A**) Antibody concentrations in serum (left panel) and ELF (right panel) based on two rabbits sacrificed at indicated time points post infusion of ASN100. Antibody concentrations are shown as group mean values +/− SEM indicating animal-to-animal variability. (**B)** The ratio of ELF and serum concentrations of ASN100 mAbs and rabbit IgG (calculated from the group means) at the indicated time points post infusion. Error bars indicate SEM.

ASN-1 and ASN-2 serum levels peaked at approximately 0.5 mg/mL at the earliest time point measured (2 hours post dosing), and then gradually decreased resulting in a half-life of more than 5 days that is in line with reports of human IgGs in rabbits^32^. ASN-1 and ASN-2 concentrations at the different time points were very similar, and the sum of the two equaled the total human IgG level. The rabbit IgG concentrations remained constant (within the range of variability among individual animals) (Fig. 5A, left panel).

The calculated ELF-concentrations of ASN-1 and ASN-2 and that of total human IgG reached peak values by 48 hours post dosing (Fig. 5A, right panel). Total rabbit IgG levels in ELF were constant throughout the 2 weeks observation period. At 24 hours post infusion, ASN100 ELF-concentrations were approximately half of their corresponding serum levels, and by 48 hours, they reached the same levels as measured in the corresponding serum samples. Thereafter, mAb levels in the ELF decreased in parallel with the serum concentrations and the ratios of ELF to serum concentrations remained constant until the end of the study (Fig. 5B).

**Figure 6:**
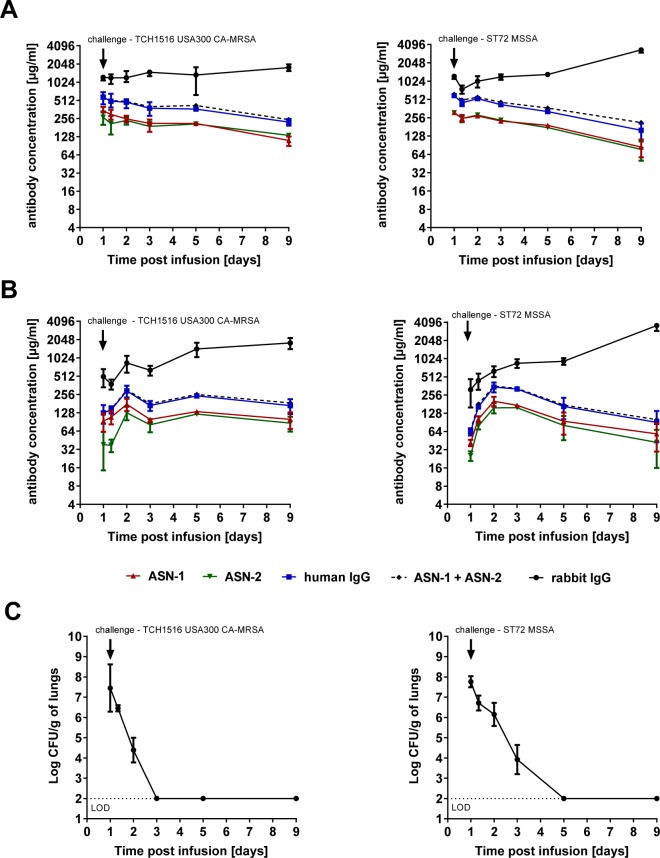
Pharmacokinetics of ASN-1 and ASN-2 in serum and ELF of *S. aureus* infected rabbits as well as the kinetics of bacterial lung clearance. Rabbits were immunized with 40 mg/kg of ASN100, 24 hours prior to lethal intra-tracheal challenge with *S. aureus* strains USA300 CA-MRSA (TCH1516) strain (left panels) or ST72 MSSA strain (right panels). Cohorts of rabbits (n = 3 per group) were sacrificed at indicated time-points. Concentrations of ASN-1 and ASN-2 as well as total human and rabbit IgG were determined in serum (**A**) and ELF (**B**) and are shown as group mean values +/− SEM. (**C**) Lung burden of treated rabbits infected with *S. aureus* strains USA300 CA-MRSA (TCH1516) (left panel) or ST72 MSSA (right panel) was determined. Group mean values +/− SEM and the lower limit of detection (LOD) are shown.

The pharmacokinetic parameters of ASN100 were also assessed in animals infected with *S. aureus*. Rabbits were passively immunized with ASN100 (same dose as for the non-infectious studies), and 24 hours later infected with lethal inocula of the USA300 CA-MRSA or the ST72 MSSA strain. Serum mAb levels showed similar kinetics to those observed in uninfected animals (Fig. 6A). However, total rabbit IgG levels increased by day 8 by an average of 1.5- and 3-fold in infected animals, which was indicative of an immune response to the *S. aureus* infection. MAb levels in the ELF peaked by 48 hours post infusion, similarly to those observed in uninfected animals (Fig. 6B). Although the kinetics were the same, the mAb and rabbit IgG concentrations were lower in the ELF of infected animals (but not in the serum). Importantly, the sum of ASN-1 and ASN-2 levels equaled that of the total human IgG, suggesting that at this ASN100 dose, there was no measurable toxin-dependent mAb depletion *in vivo*. This was observed in spite of significant bacterial load in the lungs measured in BALF until 24 or 48 hours post challenge with both, the MRSA and MSSA strain, respectively (Fig. 6C).

## Discussion

*S. aureus* pneumonia is a severe acute infection characterized by the development of marked lung inflammation and edema. It is associated with high mortality in hospitalized patients, especially those who receive mechanical ventilation and in community setting following viral infections when it can appear in fulminant form (necrotizing pneumonia). Most *S. aureus* pneumonia cases are caused by methicillin-susceptible isolates (MSSA), consequently the medical need reaches beyond the urge to find novel therapies for MRSA. In a retrospective, observational clinical study of *S. aureus* ventilator-associated pneumonia, we found that MSSA strains had a higher propensity to induce progression from airway colonization to pneumonia than MRSA strains^33^. This was observed in spite of heavy antibiotic use in this study population, which appeared to be inefficient to prevent progression to pneumonia^34^.

An increasing body of evidence supports the notion that toxins produced by *S. aureus* are involved in pneumonia pathogenesis in humans^1,33^ and in animal models^11,20,35,36^. This study shows that neutralization of six pore-forming cytotoxins of *S. aureus* is an efficacious approach to prevent mortality in rabbit models of acute, lethal pneumonia. ASN100, the combination of ASN-1 and ASN-2 human monoclonal antibodies that together neutralize six cytotoxins, elicited high prophylactic efficacy against all *S. aureus* strains tested. We used four different *S. aureus* strains, representing prevalent MRSA clonal types that cause community- and hospital-associated *S. aureus* infections including pneumonia, namely a USA300 CA-MRSA (*lukSF-PV* positive) and a USA100 HA-MRSA (*lukSF-PV* negative) isolate, and two MSSA strains, one expressing LukSF-PV, the other one not. Independent of the genetic background and the presence of the *lukSF-PV* gene, all strains caused lethal pneumonia, at comparable challenge inocula, which confirms that LukSF-PV is not indispensable for causing lethal pneumonia in rabbits. We observed full protection against the two MRSA strains with a lower dose of the antibodies (5 mg/kg ASN100), compared to the two MSSA strains (10 to 20 mg/kg ASN100), irrespective of LukSF-PV expression. Diep *et al*. previously reported the prophylactic efficacy of human IVIG preparation in similar rabbit pneumonia models^21^, showing that polyclonal human serum IgGs affinity-purified with alpha-hemolysin and LukSF-PV were sufficient for protection. Depletion of such antibodies from IVIG greatly diminished protection against the *lukSF-PV* positive USA300 CA-MRSA strain. However, depletion of IVIG with LukSF-PV also removes antibodies that are capable of binding to and neutralizing HlgAB, HlgCB and LukED, due to the high amino acid homology (up to 80%^27^) among these bi-component leukocidins, and *vice versa*, LukSF-PV can pull down cross-neutralizing antibodies. It has been elegantly demonstrated in a recent report that human memory B cells and serum antibodies exhibited cross-reactivity among the leukocidins, suggesting the targeting of conserved epitopes^37^. Importantly, *S. aureus* strains express greatly variable levels of the leukocidins (most do not express LukSF-PV) and USA300 CA-MRSA strains produce relatively low levels of the gamma-hemolysins and LukED^28^. Moreover, while rabbit phagocytic cells are highly susceptible to LukSF-PV, HlgAB, HlgCB, and LukED, their sensitivity to the fifth leukocidin, LukGH, is significantly lower compared to human phagocytes^25^. Therefore, the generalization that Hla and LukSF-PV neutralization is sufficient for protection against all *S. aureus* isolates is not justified. Even though rabbits are currently the most suitable small animal model to study *S. aureus* cytotoxins *in vivo*, the contribution to pathophysiology of all six toxins was never analyzed in a systematic manner. This could result in an over-estimation of their relevance in human disease based on data obtained in rabbits.

We further dissected the effect of toxin neutralization in the USA300 CA-MRSA model early during the infection both macroscopically and microscopically, as well as microbiologically. The severe damage to the lung tissue, development of edema and inflammation were already evident at 12 hours post-challenge. All disease parameters and bacterial burden were significantly reduced by the highest dose of ASN100 (20 mg/kg), but not reduced, or only to a lower extent, with the four-fold lower dose, despite the 100% survival of the animals. An increased number of white blood cells was observed in all animals treated with different doses of ASN100. These observations support the role of ASN100 in the neutralization of the *S. aureus* toxins responsible for tissue damage and killing of immune cells in the lungs. Additional studies to dissect the contribution of the individual toxins in these models are warranted in the future.

The efficacy data prompted us to perform pharmacokinetic (PK) studies to evaluate the penetration of the ASN100 mAbs into the lungs of uninfected rabbits. Quantification of the mAbs either individually with ASN-1 and ASN-2 specific antibodies or together with anti-human IgG reagents in BALF-samples, revealed significant amounts of ASN100 being present already at 24 hours and reaching peak levels by 48 hours following the administration of a single intravenous dose. The mAb concentrations in the epithelial lining fluid (ELF; normalized for the dilution of BALF), were approximately 50% at 24 hours and 100% at 48 hours post-immunization, and the serum-to-ELF ratios (calculated using the single matched time point PK samples) remained constant from 48 hours until the end of the experiment (14 days). Very limited information is available in the literature about the penetration of human IgG antibodies from the blood to the ELF in the lung, especially of those antibodies that do not bind to host tissues, such as anti-infective mAbs. A PK study of a human IgG1 mAb specific for the flagellin of *Pseudomonas aeruginosa* performed in uninfected mice indicated fast kinetics with peak IgG-levels in BALF by 24 hours (no calculation of actual ELF concentrations) comparable to those reported in this study in rabbits^38^. It is not understood what mechanism - active transport and/or passive diffusion - is responsible for the appearance of plasma IgGs in ELF. Interestingly, a human IgM mAb that is also specific to *Pseudomonas aeruginosa* (LPS O-antigen) was not detectable in BALF of uninfected mice, but only after bacterial challenge, suggesting that lung tissue infection and damage was required for the lung penetration of this high-molecular-weight (pentameric) mAb^39^. To assess whether such PK studies with human mAbs in animals are predictive for the human PK, comparative studies and modelling in animals and humans will be required. In this respect it is noteworthy that a small human lung penetration study with 12 healthy volunteers (embedded in a Phase 1 safety study; EudraCT #2015-003144-39) confirmed the appearance of ASN100 mAbs in the ELF during the first two days after intravenous injection and remained detectable for at least 30 days (the end of monitoring)^40^.

Importantly, in this study, we did not observe depletion of ASN100 mAbs in the serum of infected animals treated with 40 mg/kg, which translates to the fixed clinical dose of 3600 mg tested in a Phase 2 efficacy study (NCT02940626) for the prevention of *S. aureus* pneumonia in ventilated patients. Moreover, the sum of ASN-1 and ASN-2 as well as total mAb ratios in the ELF were not affected in rabbits infected intra-tracheally with *S. aureus* despite the presence of bacteria for up to 3 days post challenge. Both human and rabbit total IgG levels were lower in the BALF of infected compared to uninfected animals. One possible explanation for this is antibody depletion by the abundant IgG-binding proteins of *S. aureus*, SpA (Protein A) and Sbi (*S. aureus* binder of IgG).

*S. aureus* pneumonia in humans is certainly not as aggressive as it is in these lethal rabbit models where intratracheally applied bacteria disseminate systemically, a disease kinetics that is rarely found in humans. This might represent a caricature exaggerating the main features of *S. aureus* pneumonia. Moreover, most humans have *S. aureus* specific antibodies, especially against surface antigens^41^, which are not present in naïve animals. The high prevalence of *S. aureus* infections, however, suggests that the natural antibody levels are not sufficient to prevent disease^1,42^. Therefore, providing highly potent antibodies neutralizing six *S. aureus* toxins that compromise host defenses in a significant way, has the potential to reduce disease in high-risk individuals or ameliorate disease in therapeutic settings.

## Materials and Methods

### Bacterial strains and preparation of challenge inocula

The following *S. aureus* strains were used in these studies: USA300 CA-MRSA strains TCH1516 (ST8-IV-t622, *lukSF-PV*+ from ATCC^®^ BAA-1717^TM^) or LAC (ST8-IV-t008, *lukSF-PV*+; kindly provided by Frank DeLeo (NIAID, Bethesda, MD, USA); the USA100 HA-MRSA strain (ST5-II-t002, *lukSF-PV*−) and the *lukSF-PV*− MSSA strain (ST72-t148) were collected from mechanically ventilated patients^33^; the *lukSF-PV*+ MSSA strain (ST152-t3621) was kindly provided by Francois Vandenesch (Université Claude Bernard Lyon 1, INSERM 1111, Lyon, France). The genotypes of the different isolates are depicted in Supplementary Table 1. Bacterial strains were maintained and cultured using standard microbiological conditions. Bacterial challenge inocula were prepared according to established protocols at Vivexia (Dijon, France) or Fidelta Ltd. (Zagreb, Croatia). Mid-log phase cultures were obtained in BHI (Fidelta) or CCY medium supplemented with 10% pyruvic acid (Vivexia), washed twice and diluted to the desired concentration in sterile saline prior to challenge.

### Animal welfare and monitoring

Animal experiments were approved by the IACUCs, as well as the respective competent authorities and performed at Vivexia (C2EA grand campus Dijon N°105, Dijon, France approved by the Ministère de l’enseignement supérieur, de la recherche et de l’innovation, approval number: APAFIS#5606-2016060210418843 v3) or Fidelta Ltd. (Committee on Animal Research Ethics (CARE-Zg; CAREZG_15-07-23_57), Fidelta Ltd. Zagreb, Croatia approved by the Ministry of Agriculture, Veterinary and Food Safety Directorate, Republic of Croatia, approval number: KLASA: UP/I-322-01/15-01/103, URBROJ: 525-10/0255-16-7). All methods were performed *lege artis* in accordance with European and national guidelines and regulations. Male New Zealand White SPF rabbits were obtained from the Zootechnical Center (approval number: N° C 21 464 04 EA, University of Burgundy, Dijon, France) or Charles River (France). Food and water were provided *ad libitum* throughout the study. Health was monitored according to good veterinarian practice and institutional guidelines. Moribund animals, fulfilling the humane endpoints were euthanized prior to intended study end, all other animals at study end by ketamine/xylazine sedation and lethal pentobarbital injection.

### Efficacy studies

Rabbits with starting body weights of approximately 3 kg were randomized in the respective study cohorts. ASN100 (0.08 to 20 mg/kg) or placebo was administered intravenously in a single-bolus (1 mL/kg). 24 hours post infusion, animals were infected with lethal bacterial inocula between 1.5 to 9 × 10^9^ CFU/animal, *via* a tracheal catheter. Survival of animals was monitored every 3 hours within the first 2 days followed by daily monitoring up to day 7 post challenge. For histopathological studies, animals were euthanized at 12 hours post infection.

### Macroscopic scoring and lung-edema rate

Pulmonary injury was scored and calculated as previously described^43,44^. The total macroscopic score is the sum of scores for individual pulmonary lobes (scoring grid: 0 = normal; 1 = scar; 2 = slight congestion; 3 = red congestion; 4 = grey congestion; 5 = yellowish congestion), with 1 additional point per side (left or right) for each, purulent pleural effusion and presence of gangrene. The individual lung edema rate was evaluated by calculating the lung-weight-to-body-weight ratio (LW/BW × 10^3^). The total lung weight corresponds to the cumulative weight of all the individual pulmonary lobes.

### Histopathology and bacterial burden in lungs, spleens and kidneys

Following euthanasia and macroscopic scoring, the spleen, kidneys as well as the individual pulmonary lobes were aseptically excised. 5 mm slices were sampled for all organs (a transverse slice at the hilum level was sampled from kidneys), fixed in 10% neutral buffered formalin and sent for blinded histopathology evaluation by a veterinarian pathologist (Histalim, Montpellier, France). Histopathological pulmonary scores, macrophage- and neutrophil-counts were evaluated based on hematoxylin and eosin (H&E) stained tissues according to standard pathological scoring using semi-quantitative qualifiers (*i.e*. focal, multifocal, locally extensive or diffuse) and an inflammation-score applying a severity scale: 0: within physiological limits, 1: minimal, 2: mild, 3: moderate, 4: marked, 5: severe.

Lung, spleen, and kidney samples were weighed and homogenized. Serial 10-fold dilutions of organ-homogenates were plated and bacterial-counts were determined following incubation. Bacterial-loads in each lung lobe (expressed as mean pulmonary bacterial load), kidney, and spleen were expressed as logCFU/g of each organ.

### Sampling for the pharmacokinetic analysis of ASN100 in uninfected and *S. aureus-*infected rabbits

For all studies, pre-immune sera were collected from individual rabbits 24 hours prior to mAb-infusion.

Serum PK studies: ASN100 was intravenously administered at 40 mg/kg and control animals received isovolumetric amounts of placebo (n = 3 rabbits/group in two independent studies). Sera were collected immediately at the end of infusion (EOI) as well as at 1, 2, 3, 4, 5, 6, 12, 24 hours and once daily between study days 2 and 14 post infusion. In infectious studies, rabbits were intubated and infected with lethal inocula (9 × 10^9^ CFU/rabbit) of either the USA300 CA-MRSA (TCH1516) or the ST72 MSSA strains at 24 hours post ASN100 infusion. Serum was collected at 22 hours post infusion, immediately after challenge, at 1, 2, 3, 4, 5, 6, 12, 24 hours and once daily between study days 2 and 14 post challenge.

ELF-PK studies: ASN100 was intravenously administered at 40 mg/kg and control animals received isovolumetric amounts of placebo. Cohorts of uninfected rabbits (n = 2/group) were sacrificed 2 hours after EOI or on days 1 to 5, 10 and 14 post infusion, where samples were collected. Blood was drawn from the lateral ear-vein and sera prepared. BALF was collected after intra-tracheal intubation and instillation of a single bolus (2 mL/kg) of sterile PBS (37 °C), followed by centrifugation (3500 rpm; 5 min) All samples were stored frozen (−80 °C) until further testing.

In infectious studies, rabbits (n = 3/group) were intubated and infected with lethal inocula of either the USA300 CA-MRSA (TCH1516) or the ST72 MSSA strains at 24 hours post ASN100 infusion. Serum was collected at 22 hours post infusion. Serum and BALF was collected immediately after challenge, 8, 24 and 48 hours as well as on days 4 and 8 post challenge. In parallel, cohorts of 3 placebo control animals, which were expected to succumb to infection within 48 hours, were sacrificed at early stages of infection. CFUs were determined in lungs of infected animals at all sampling time-points and were expressed as logCFU/g of lungs.

### Monoclonal antibodies

ASN-1 and ASN-2 were produced by stably transfected CHO cell lines and purified, as previously described^28^.

### Determination of antibody levels in serum and BALF samples

Antibody concentrations in serum and BALF were measured in quantitative sandwich ELISA assays. All assays were performed in 96-well MaxiSorp^TM^ plates (Nunc) using TMB (ThermoFisher Scientific) as HRP substrate for detection. Anti-ASN-1 or anti-ASN-2 anti-idiotype antibodies in Fab-A-FH format (BioRad) were used as capturing reagents to bind free ASN-1 and ASN-2 in serum and BALF. HRP-conjugated F(ab’)_2_ fragment rabbit anti-human IgG (Fcγ-fragment specific, Jackson Immuno Research, #309-036-008) were used for detection. ASN-1 and ASN-2 study material was used for generation of calibration standards in these assays. Sensitivity and lack of cross-detection was evaluated and confirmed with ASN-1 and ASN-2 spike-in samples generated in rabbit serum. Total human IgG (total ASN-1 + total ASN-2) concentrations were measured using Fcγ-fragment specific rabbit anti-human F(ab’)_2_ fragment (Jackson Immuno Research, #309-006-008) for capture and HRP conjugated rabbit anti-human F(ab’)_2_ fragments (Jackson Immuno Research #309-035-006) for detection. Standard curves were prepared with an equimolar mixture of ASN-1 and ASN-2. Total rabbit IgG levels were assayed using a light chain specific, monoclonal mouse anti-rabbit IgG (Jackson Immuno Research, #211-002-171) in combination with an HRP conjugated donkey anti-rabbit IgG (H + L) F(ab’)_2_ fragments for detection (Jackson Immuno Research, # 711-036-152). Selectivity of human and rabbit IgG assays was confirmed with purified human and rabbit IgGs and naïve sera. Sample dilution was dependent on the mg/kg dose group and adjusted to allow raw data collection in the ng/mL working range for all assays and analytes. Data were analyzed in a 4-Parameter Logistic (4PL) regression model using Prism^®^ 6.07 (GraphPad). To normalize the dilution bias resulting from the BALF collection and to obtain antibody ELF concentration estimates, urea was measured in serum and BALF using a commercial urea assay kit (Abcam ab83362) according to manufacturer’s instructions. BALF IgG concentrations were multiplied by the ratio of serum to BALF urea levels in order to obtain dilution-corrected antibody concentrations.

### Bacterial culture supernatant generation

*S. aureus* culture supernatants (CS) were generated in *(i)* CCY: 3% yeast extract (Fisher BioReagents) supplemented with 2% bacto-casamino acids (Amresco), 2.3% sodium pyruvate (Fisher BioReagents), 0.63% Na_2_HPO_4_ (Fisher BioReagents) and 0.041% KH_2_PO_4_ (Sigma), *(ii)* BHI (Fluka) and *(iii)* RPMI-CAS (RPMI-1640 medium (Gibco) supplemented with 1% casamino acids (Amresco). Overnight cultures obtained from a single colony were diluted to OD_600nm_ = 0.03 in fresh culture medium and grown to early stationary phase for 8 hours. All tested *S. aureus* strains reached similar bacterial densities in the respective media. CS were generated by centrifugation (5000 × g, 4 °C), followed by filter sterilization (0.1 µm pore size PVDF syringe filters; Millipore).

### Immunoblotting to detect cytotoxin expression

*S. aureus* CSs were analyzed by SDS-PAGE (4–20% gradient gel, BioRad, #456-1096) under reducing conditions. Proteins were transferred to PVDF (Hla, LukG and LukS-PV) or nitrocellulose membranes (LukD, HlgB) (Trans-Blot Turbo device, BioRad). Hla, LukD, and HlgB were detected using monospecific human IgGs, LukS-PV with a monoclonal mouse antibody (IBT Bioservices) and LukG expression using a rabbit anti-LukB (LukG) polyclonal antibody (IBT Bioservices) as described previously^28^. Recombinant toxins expressed in *E. coli*^28^ were used as positive controls.

### Statistical data analyses

Group comparison and survival were statistically analyzed using Prism^®^ 6.07 (GraphPad). Methods used for each dataset are indicated in the respective figure legends.

## Supplementary information

Supplementary Information

## Data Availability

The material and reagents used, datasets generated and/or analyzed during the current study are available from the corresponding author on reasonable request.
