# Supplementary material for: Preventing lung pathology and mortality in rabbit Staphylococcus aureus pneumonia models with cytotoxin-neutralizing monoclonal IgGs penetrating the epithelial lining fluid
Source: Sci Rep. 2019 Mar 29;9:5339. doi: 10.1038/s41598-019-41826-6 (PMC6441091; doi:10.1038/s41598-019-41826-6)

Preventing lung pathology and mortality in rabbit *Staphylococcus aureus* pneumonia models with cytotoxin-neutralizing monoclonal IgGs penetrating the epithelial lining fluid

Lukas Stulik[1,#,*], Harald Rouha[1,#], Delphine Labrousse[2], Zehra Claire Visram[1], Adriana Badarau[1], Barbara Maierhofer[1], Karin Groß[1], Susanne Weber[1], Miroslava Dominis Kramarić[3], Ines Glojnarić[3], Gábor Nagy[1,+], Delphine Croisier[2], Eszter Nagy[1,+]

[1] Arsanis Biosciences, Vienna, Austria; [2] Vivexia, Dijon, France; [3] Fidelta Ltd., Zagreb, Croatia

[#] L. S. and H. R. contributed equally to the work

[+] Independent researcher, Vienna, Austria

* Correspondence and requests for materials should be addressed to

Lukas Stulik, PhD

Arsanis Biosciences

Helmut-Qualtinger-Gasse 2, 1030 Vienna, Austria

P: +43-1-7990-117-24, F: +43-1-7990-117-99

E-mail: lukas.stulik@arsanis.com

Figure S1

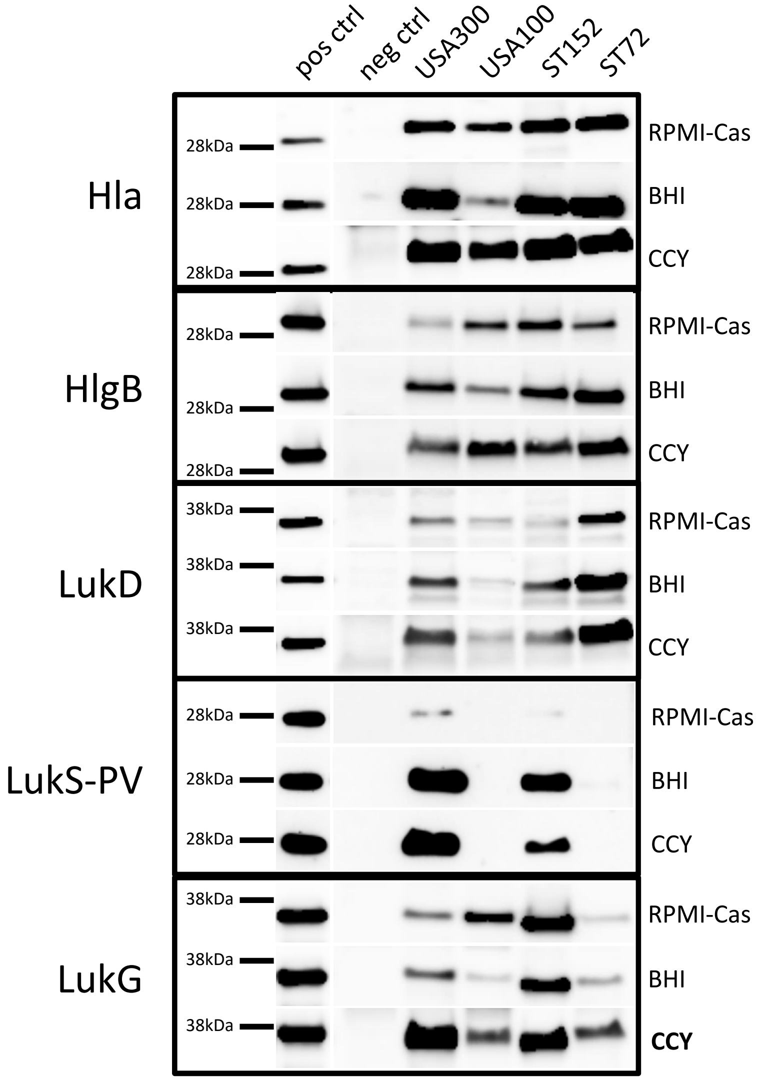

Figure S1. Toxin expression profile of *S. aureus* strains. Immunoblot analysis for the detection of Hla and the bi-component toxins in the culture supernatant of a prototype USA300 CA-MRSA (TCH1516), a USA100 HA-MRSA, and two MSSA strains with ST152 and ST72, grown in RPMI-CAS, BHI or CCY growth media. An isogenic *S. aureus* mutant strain lacking the genes expressing Hla and all bi-component leukocidins (TCH1516Δhla/hlgACB/lukGH/lukED/lukSF-PV) was used as an assay control (neg ctrl). Pos ctrl: recombinant toxins (20 ng/lane for LukG and LukD, 50 ng/lane for HlgB and Hla and 100 ng/lane for LukS-PV). Figure assembled from individual blots as shown in the Supplementary Information file.

Supplementary Table 1

Table S1: *S. aureus* strains used in this study

Strain Name	MLST-SCCmec-spa-type	MRSA/MSSA	lukSF-PV	lukED	hlgACB	lukGH	hla	Source of strain
USA300 CA-MRSA TCH1516	ST8-IV-t622	MRSA	+	+	+	+	+	ATCC® BAA-1717™
USA300 CA-MRSA LAC	ST8-IV-t008	MRSA	+	+	+	+	+	Dr. Frank DeLeo (NIAID)
USA100 HA-MRSA	ST5-II-t002	MRSA	-	+	+	+	+	[33]
ST152 MSSA	ST152-t3621	MSSA	+	+	+	+	+	Dr. François Vandenesch (INSERM)
ST72 MSSA	ST72-t148	MSSA	-	+	+	+	+	[33]

Supplementary Information 1

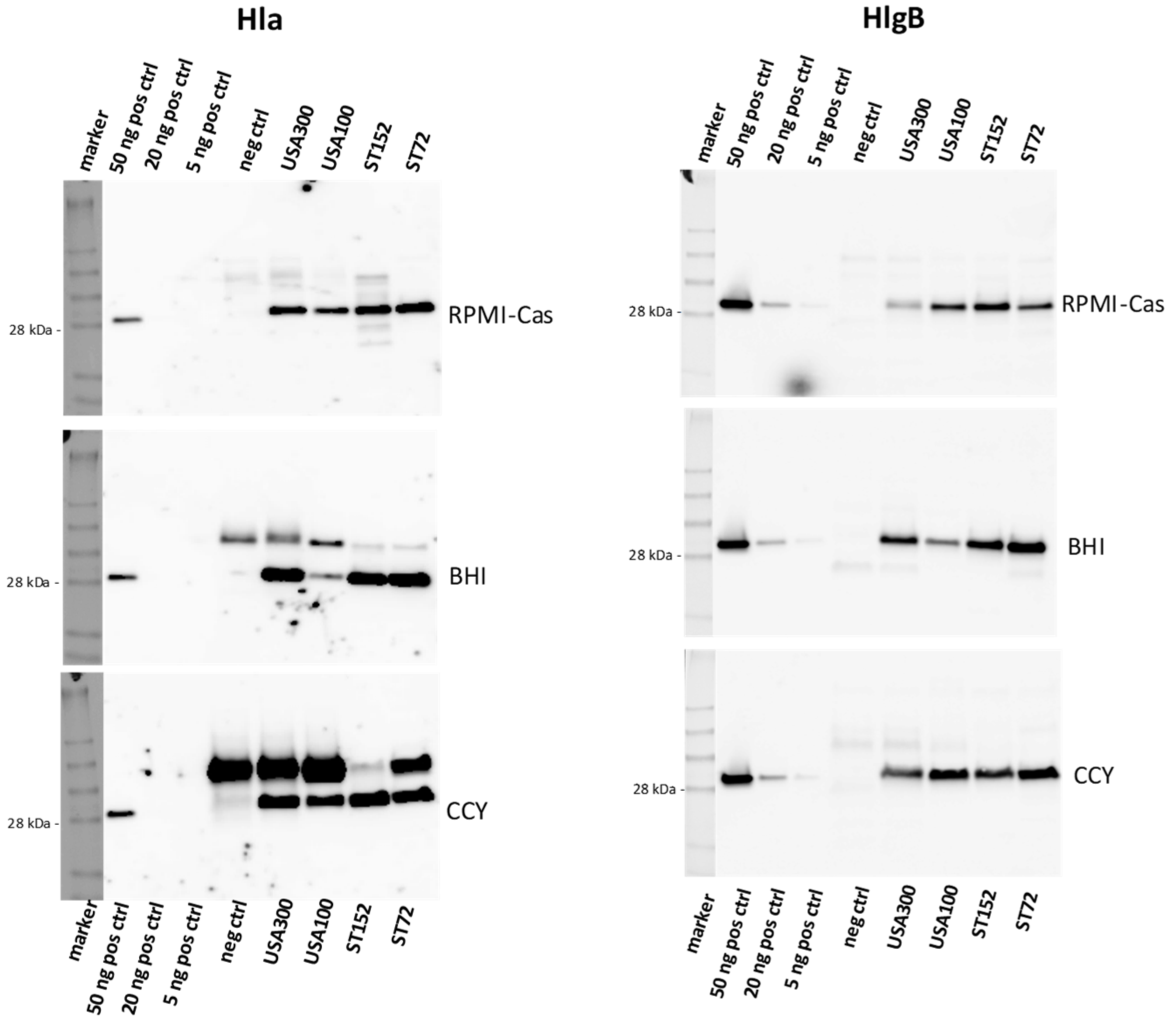

Supplementary Information 2

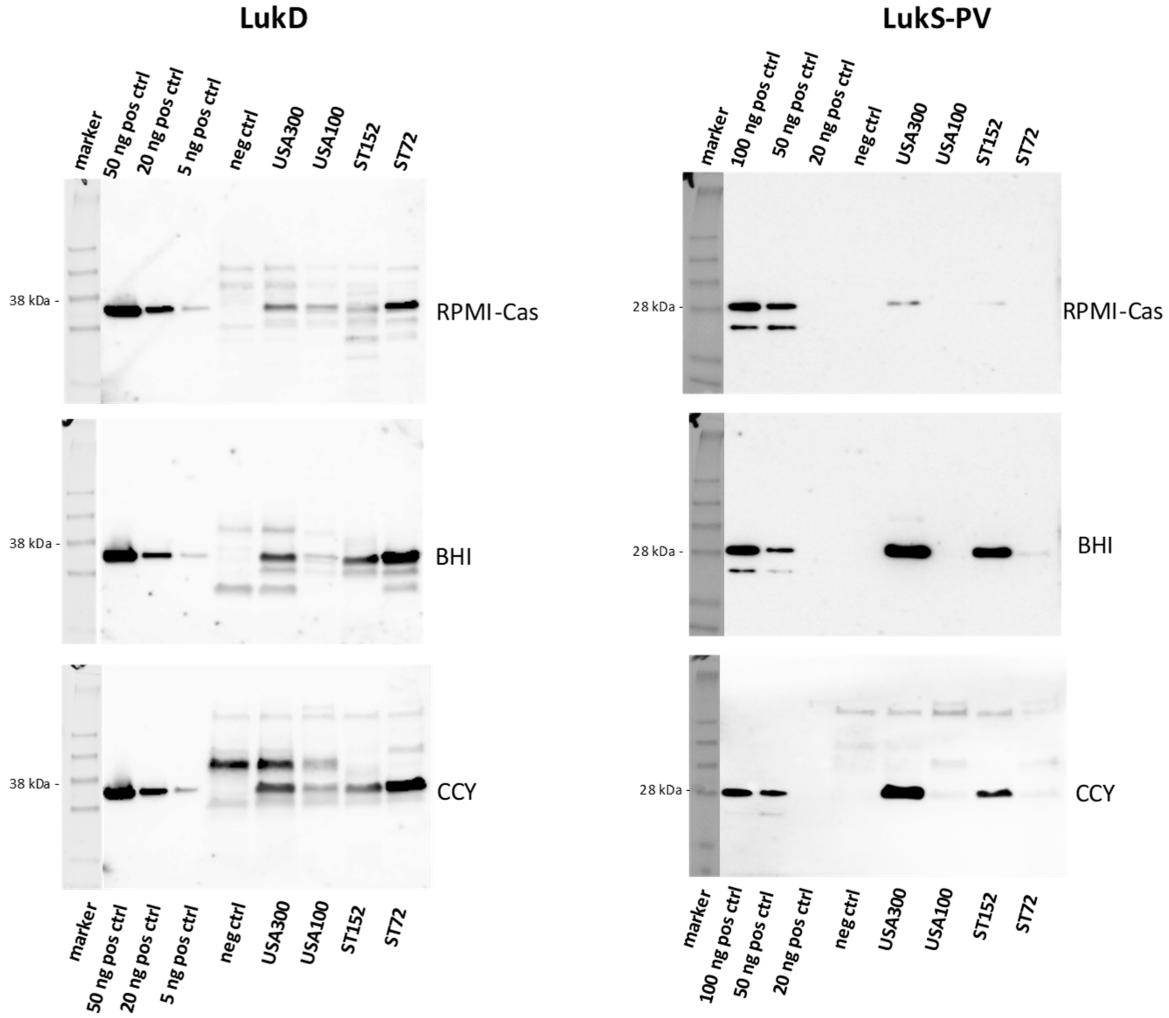

Supplementary Information 3

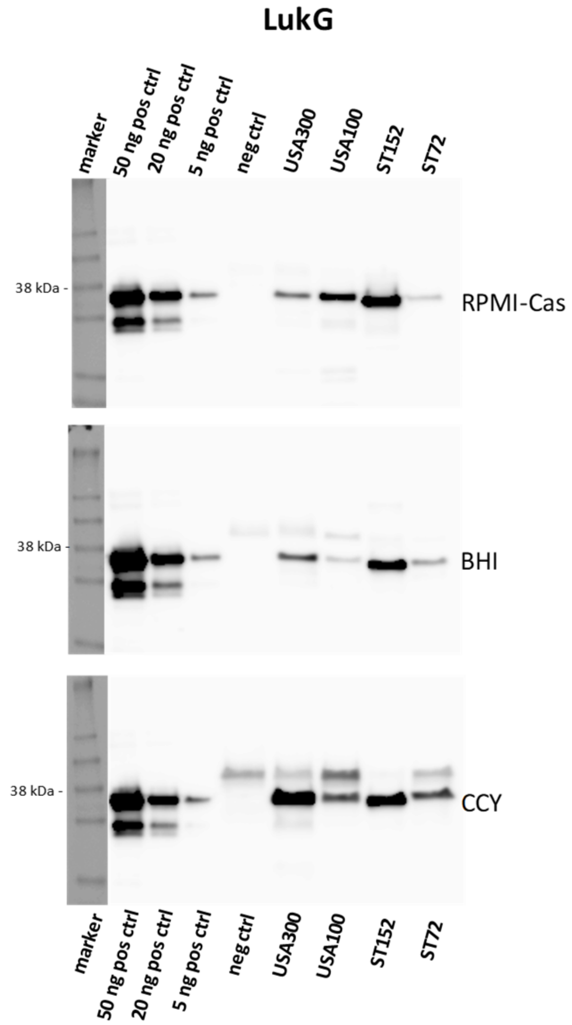

Supplement: Supplementary file 1 — Supplementary Information [file 41598_2019_41826_MOESM1_ESM.pdf]
